# Comparative Studies for the Fabrication of WTe_2_ Nanoribbons in the Tellurization Process

**DOI:** 10.1021/acs.jpcc.5c08745

**Published:** 2026-05-11

**Authors:** Jeļena Sušinska, Aina Semjonova, Reinis Ignatans, Gunta Kunakova

**Affiliations:** † Institute of Chemical Physics, Faculty of Science and Technology, 61769 University of Latvia, Riga LV-1004, Latvia; ‡ Department of Chemistry, Faculty of Medicine and Life Sciences, 61769 University of Latvia, Jelgavas Street 1, Riga LV-1004, Latvia; § Institute of Solid State Physics, 61769 University of Latvia, Ķengaraga Street 8, Riga LV-1063, Latvia

## Abstract

Tungsten ditelluride is a layered semimetal with higher-order topological insulator properties and robust one-dimensional (1D) states along certain crystallographic axes, making it promising for future spintronic applications. While bulk and exfoliated materials have been extensively studied, WTe_2_ nanoribbons and nanowires offer advantages for probing edge-state physics due to their enhanced surface-to-volume ratio and confined geometry. However, existing growth approaches for producing WTe_2_ nanostructures struggle to yield materials of sufficient quality. In this work, we investigate the growth and charge transport characteristics of WTe_2_ nanoribbons (NRs) synthesized through the tellurization of WO_3_ nanoribbons, comparing how different precursor compositions, WO_3_ with and without a NaCl:Te additive, alter the resulting material properties. The tellurization of the ribbons obtained using a NaCl:Te additive allows the production of highly crystalline WTe_2_. Charge transport measurements for individual WTe_2_ nanoribbon four-terminal devices indicate that surface capping is essential to prevent rapid oxidation. This study establishes a pathway for the fabrication of freestanding WTe_2_ nanoribbons of different geometrical parameters via the tellurization process.

## Graphical abstract

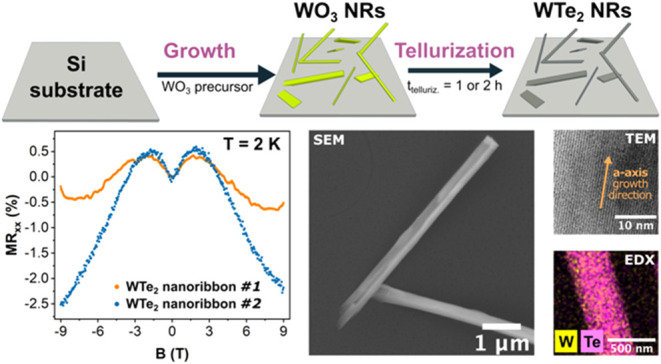

## Introduction

Tungsten ditelluride (WTe_2_) is a transition-metal dichalcogenide (TMD) with distinctive electronic properties, including two-dimensional (2D) topological insulator (TI) states in monolayer^1,2^ and large nonsaturating magnetoresistance with nearly perfect charge compensation in few-layer and bulk materials.^3^ The exploitation of these nonconventional properties is strongly dependent on the material quality and dimensions. Recently, it was demonstrated that bulk WTe_2_ exhibits higher-order topological insulator (HOTI) behavior,^4^ with protected one-dimensional (1D) states that are conductive and robust against scattering. Additionally, helical hinge states in WTe_2_ have been proposed,^5^ and evidence of conducting states likely representing these helical hinge states localized at the sample edges has been demonstrated in the ribbon-shaped, etched WTe_2_ thin structures. The reported states are observed only along the *a*-crystallographic axis of the etched ribbon sample while being delocalized along the *b*-axis.^6^ Furthermore, studies of 2D topological insulator properties that probe edge-state transport phenomena require WTe_2_ in the form of thin ribbons. All of the experimental studies so far have been conducted on exfoliated flakes or on flakes etched into ribbon-shaped structures, producing an optimal edge geometry. Chemical etching and lithography processing may favor the formation of charge puddles, which can obscure edge-state transport, as recently noted for other 2D topological insulator material systems.^7^ Materials with as-grown ribbon shapes are significant in harnessing edge-state phenomenology.

A wide range of methods for obtaining WTe_2_ nanowires (NWs), nanobelts, and nanoribbons (NRs) has been investigated in prior studies. One demonstrated method is based on the eutectic alloy-assisted approach,^8^ where W/Cu films were predeposited and tellurized to obtain WTe_2_ nanobelts. 1D WTe_2_ nanostructures can also be fabricated via top-down exfoliation from bulk crystals. For instance, flat and parallel-aligned WTe_2_ nanoribbons can be produced using thermal release tape (TRT), polyvinylpyrrolidone (PVP) as the anticontaminating layer, and gold as the adhesive layer.^9^ Another approach for the fabrication of 1D structures involves two steps: (1) growth of WO_3_ structures via solvothermal,^10^ hydrothermal,^11^ or thermal evaporation of WO_3_ precursor^12,13^ and (2) conversion (tellurization) to WTe_2_.^10–12,14^ Recent progress in the development of growth protocols for obtaining WTe_2_ nanoribbons and nanowires is summarized in Table S1. Among all of the methods, chemical vapor deposition (CVD) emerges as one of the most popular and versatile techniques for the bottom-up production of nanostructures, with the advantage of controlling their dimensions.^8,10–12,15–19^

Despite significant efforts to develop growth protocols for WTe_2_ nanowires and nanoribbons, the reported charge transport data in most cases show insulating or wide band gap semiconductor characteristics.^12,19^ Rapid formation of native oxide governing band-bending effects may obscure pristine characteristics of WTe_2_.^20,21^ Challenges in obtaining reliable and reproducible data on the charge transport characteristics of a single WTe_2_ nanowire or ribbon persist due to poor material quality and their rapid oxidation under atmospheric conditions. Further improvements in growth methods are essential to produce high-quality, freestanding WTe_2_ nanowires or nanoribbons, minimizing destructive steps during transfer and enabling more reliable measurements. This could be achieved by improving the tellurization efficiency through comparative studies of different precursors.

In this work, we have developed growth methods for obtaining freestanding WTe_2_ nanowires and nanoribbons of different geometrical parameters, and with the crystallographic growth direction along the *a*-axis, instrumental for probing the 1D edge-state transport toward future applications in topological nanoelectronics, where nanomaterials of well-defined geometry are among the most important building blocks. By studying two approaches for the fabrication of WTe_2_ in the tellurization process, it was determined that uniform and highly crystalline nanoribbons, free from grain boundaries, can be achieved when sodium is used as a tellurization promoter. Measured resistance–temperature dependences for both as-grown and ZnO-encapsulated WTe_2_ nanowires and nanoribbons show metal-like characteristics, and negative magnetoresistance at low temperatures is likely due to increased disorder.

## Methods and Experimental Details

WTe_2_ nanoribbons and nanowires were obtained by a two-step CVD using a two-zone tube furnace (OTF-1200X-80-II). The first step involved WO_3_ nanoribbon growth, and the second was the tellurization to WTe_2_.

### Growth of WO_3_ Nanoribbons from WO_3_/NaCl/Te Precursor

A 0.07 g portion of the WO_3_/NaCl/Te (8:1:35) homogeneous mixture was placed in an 80 × 20 × 10 mm^3^ quartz boat, and a 20 × 10 mm^2^ Si[100] substrate was placed inclined with the polished side facing the precursor. A quartz boat was then placed in the tube furnace’s right zone (Figure S1). The system was purged with Ar, then pumped down to 6 mTorr; Ar was set to flow at 80 sccm, bringing the system pressure to 0.03 Torr. The synthesis was carried out at 850 °C and held for 5 min. Subsequently, the system was left to cool to room temperature.

### Growth of WO_3_ Nanowires from the WO_3_ Precursor without an Additive

A quartz boat with 0.1 g of WO_3_ powder (STREM Chemicals Inc., 99.99%) was placed in a ⌀ ∼ 2.5 cm quartz test tube with 10 × 10 mm^2^ Si[100] substrates located 13–15 cm downstream. The quartz tube was positioned inside the tube furnace, ensuring that the WO_3_ powder was in the furnace’s left zone and the substrate in the right zone. The system was purged with Ar several times. At the beginning of the synthesis, the pressure was set to 0.3 Torr, and hydrogen flowed at ∼30 sccm during the synthesis. The temperature of the left zone was set to 950 °C, while the temperature of the right zone was ∼580 °C. After 2 h, the system was left to cool down to room temperature.

### Tellurization

A quartz boat containing a substrate with previously synthesized WO_3_ nanoribbons and 0.15 g of Te powder (Alpha Aesar, 99.999%), positioned 5 mm upstream from the substrate, was placed inside a quartz test tube (⌀ ∼ 3 cm). The test tube was placed in the right zone of the tube furnace (Figure S1). The system was purged with Ar several times, then filled with Ar to ∼780 Torr. The synthesis was carried out at ambient pressure with an Ar/H_2_ (80/40 sccm) flow. Both zones were heated for 24 min to 600 °C, held for 1 h (nonadditive WO_3_ NRs) or 2 h (NaCl:Te additive NRs), and then left to cool down to room temperature. The hydrogen was introduced into the system when the furnace temperature reached 400 °C and switched off during the process of cooling when the temperature decreased to 400 °C.

### Characterization

The structure, morphology, and chemical composition of the nanoribbons were characterized by optical microscopy, scanning electron microscopy (SEM) with energy-dispersive X-ray spectroscopy (EDX), atomic force microscopy (AFM), transmission electron microscopy (TEM), and X-ray diffraction (XRD) analysis. As WTe_2_ is sensitive to air exposure and oxidizes rapidly, after tellurization, the sample was placed in a closed inert atmosphere to ensure short-term preservation, while the atomic layer deposition (ALD) instrument was set up. A three-nanometer ZnO layer was deposited on freestanding WTe_2_ nanoribbons, resulting in a core–shell structure. Before establishing direct metal–nanoribbon contact, samples were etched using a 1 M HCl/1 M HNO_3_ (3:1) solution.

## Results and Discussion

Tungsten ditelluride 1D nanostructures, nanowires (NWs) and nanoribbons (NRs), are synthesized using a two-step chemical vapor deposition (CVD). More details on the synthesis configuration are provided in the Supporting Information (SI), Figure S1a,b. As shown in the schematic in Figure 1a, the fabrication of WTe_2_ nanoribbons begins with the initial growth of tungsten trioxide (WO_3_) nanoribbons. We have employed two methods for growing WO_3_ nanoribbons: one with Te:NaCl as an additive to the WO_3_ powder and another without any extra additives. The obtained WO_3_ nanostructures were subsequently used in the tellurization process, carried out at ambient pressure with an Ar/H_2_ gas flow at 575 °C, where tellurium first reacts with hydrogen to form H_2_Te (synthesis configuration in SI, Figure S1c). This intermediate then interacts with the WO_3_ nanoribbons, leading to the formation of WTe_2_.^22^ SEM images of WTe_2_ nanoribbons fabricated from the corresponding WO_3_ nanoribbons grown using the WO_3_/NaCl/Te (8:1:35) precursor are depicted in Figure 1c,d. The nanoribbons exhibit a faceted structure with a “single wire growth pattern”, where each ribbon is rooted on the Si[100] substrate (Figure 1d, inset). Thinner nanoribbons are obtained from the region of the sample further from the precursor during the WO_3_ nanoribbon growth process, as the substrate was placed inclined toward the precursor (see SI, Figure S1a for details on the sample placement with respect to the powder and Figure S2a,b for the geometrical characterization of the tellurized nanoribbons). The width of the nanoribbons (*w*_NR_) ranges from several microns to around 30 nm, lengths (*L*) from 50 to 1 μm, and thicknesses (*t*_NR_) from 350 to 30 nm. The inclined placement of the Si substrate leads to a spatial gradient in precursor delivery. Consequently, a lower mass flux of precursor occurs farther from the WO_3_:NaCl source and thinner nanoribbons are obtained.

**Figure 1:**
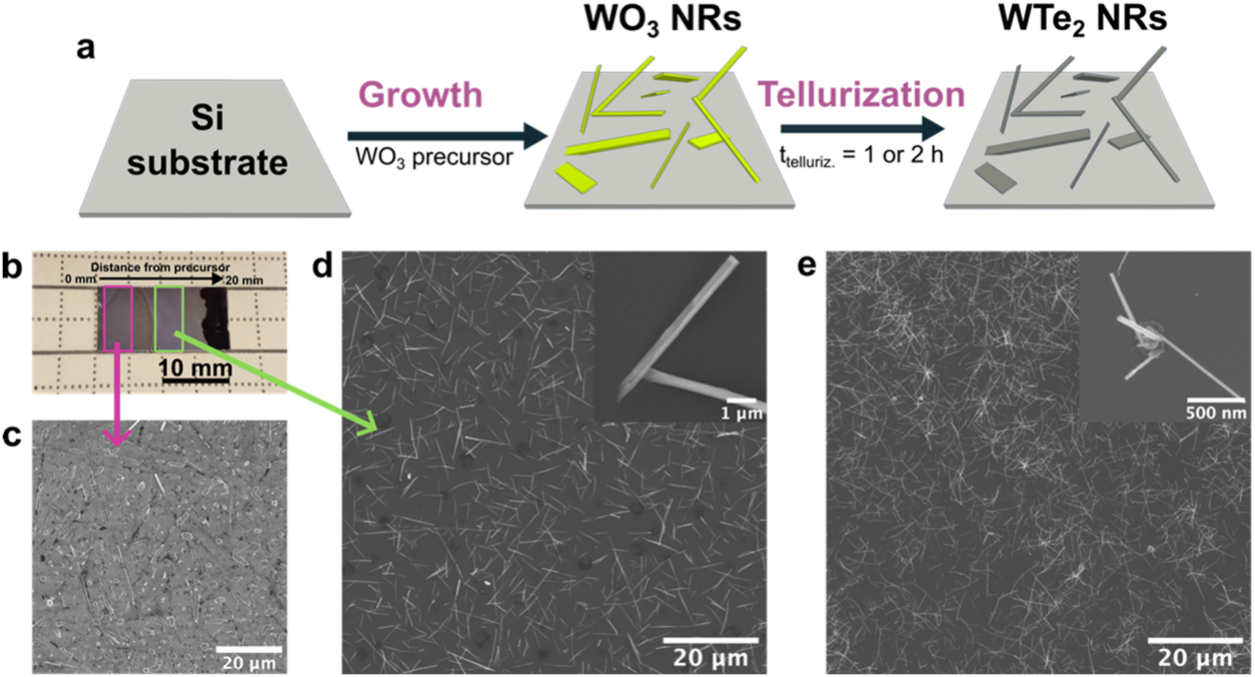
Fabrication and characterization of freestanding WTe_2_ nanoribbons. (a) Schematic representation of WTe_2_ NRs via two-step CVD. (b) Photograph of the WTe_2_ NRs sample obtained via the WO_3_:NaCl:Te method. (c) SEM image taken from the substrate area marked by the pink rectangle in (b). (d) SEM image taken from the sample’s area marked by the green rectangle in (b). The inset shows the faceted structure of the NRs. (e) SEM image of WTe_2_ NWs fabricated from WO_3_ NWs grown from WO_3_ without additives. The inset shows the higher-magnification SEM image.

WTe_2_ nanowires were also fabricated from WO_3_ nanowires obtained without using the NaCl:Te additive. In this method, both the WO_3_ powder and the Si[100] substrate were placed in a test tube (schematics in SI, Figure S1b). In contrast to the first method, the nanowires grown from the WO_3_ powder display a bouquet-like arrangement, uniformly dispersed on the substrate (Figure 1e). These nanowires originate from solid droplet-like structures, suggesting a noncatalyst vapor–liquid–solid (VLS) growth mechanism. Larger droplets or droplet clusters lead to bouquet-like growth, while smaller ones result in the formation of single nanowires. The nanowires synthesized in this approach have a rectangular cross section, but their width is about 100 times narrower than that of the nanoribbons obtained from the WO_3_:NaCl:Te. The determined geometrical characteristics for these nanowires are as follows: *w*_NW_ = 20–60 nm, *t*_NW_ = 10–50 nm, and *L* = 5–10 μm. Although the structures are not cylindrical in shape, they are referred to as nanowires in this work.

The stoichiometry of the resulting WTe_2_ nanoribbons was initially verified using SEM-EDX spectroscopy to analyze both W and Te L-series signals. It was found to depend on the distance between the WO_3_ nanowire substrate and Te powder used in the tellurization process (schematics in SI, Figure S1c). The closest W/Te ratio of 1:2 was achieved when the WO_3_ nanoribbon sample was placed 5 mm downstream from the Te powder (Figure 2a). Due to the small dimensions of the nanowires compared to the nanoribbons, EDX analysis could not be used to determine their chemical composition.

**Figure 2:**
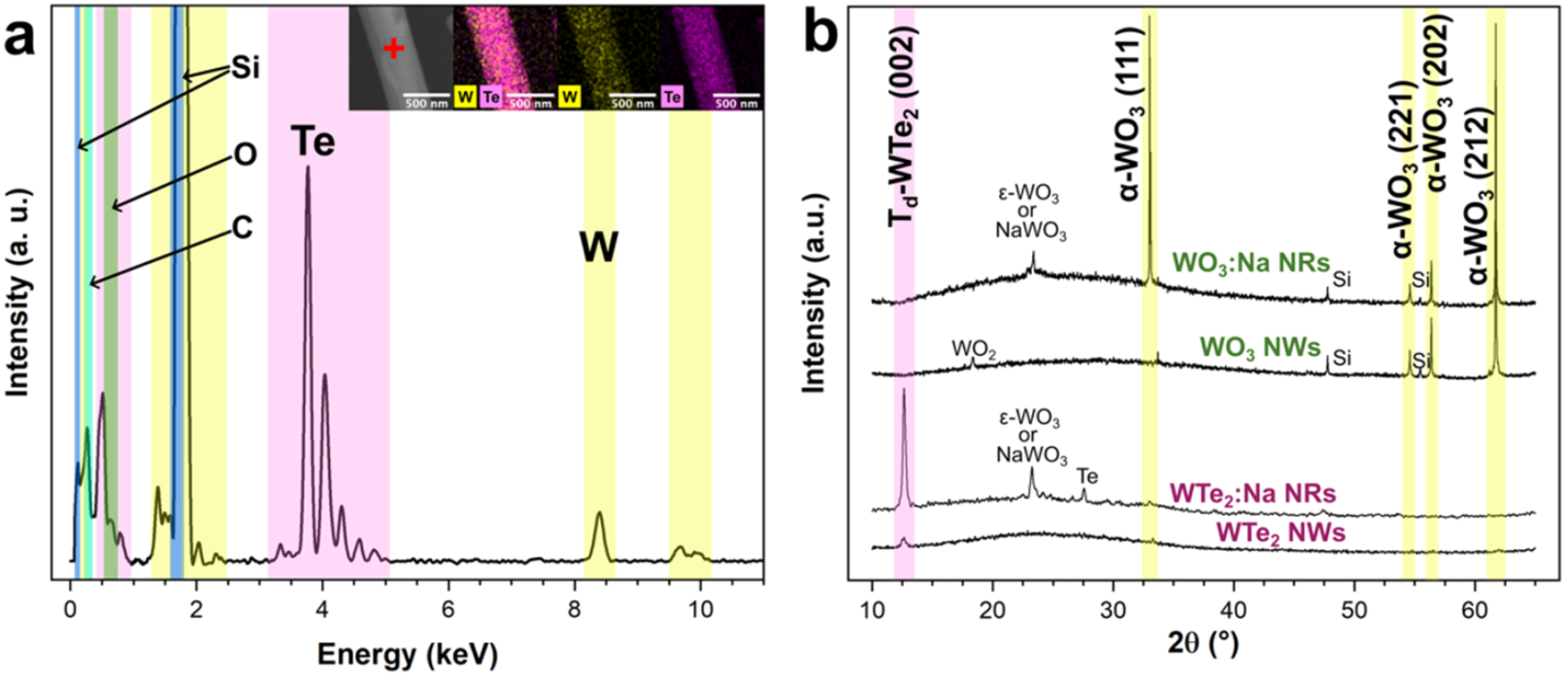
(a) EDX point spectra of a WTe_2_ nanoribbon (from the WO_3_:NaCl:Te growth approach) and mapping of W and Te elements in the inset. (b) XRD patterns of WO_3_ and the corresponding WTe_2_ nanoribbons (NRs) and nanowires (NWs).

To further characterize the WTe_2_ nanoribbons and nanowires, X-ray diffractograms were recorded. Figure 2b summarizes four spectra: two for WO_3_ nanoribbons and nanowires fabricated from WO_3_:NaCl:Te and WO_3_, respectively, and two for the corresponding WTe_2_ nanowire and nanoribbon samples.

WO_3_ nanoribbons obtained via both methods exhibit the tetragonal (α-WO_3_) phase, with characteristic peaks at 54.6, 57.7, 61.8, and 76.5° 2Θ corresponding to the (221), (202), (212), and (401) planes (Figure 2b), in agreement with the PDF 00-005-0388.^23^ The WO_3_ nanowire sample fabricated from the WO_3_ precursor without additives shows an additional peak at 33.6° 2Θ for the (002) plane and a peak at 18.3° 2Θ, which could be identified as monoclinic WO_2_ (PDF 01-082-0728 ^24^). In contrast, WO_3_ nanoribbons produced from the WO_3_:NaCl:Te did not display these two peaks; instead, a notable signal at 33.1° 2Θ was observed, correlating to the (111) plane of α-WO_3_. Additionally, these nanoribbons exhibited two peaks at 23° 2Θ, which could be associated with either the monoclinic ε-WO_3_ or NaWO_3_ (PDF 01-087-2402,^25^ PDF 01-074-2270 ^26^). Since the XRD measurements were taken over the whole area of the sample rather than a single ribbon, the presence of sodium within the nanoribbon cannot be confirmed. The XRD data for the additional samples with different precursor ratios (10:1, 8:1, 3:1, and 1:1) are summarized in Figure S5 of the SI. As the sodium concentration increases, the signals from the tungsten bronze phase appear more distinctively.^27^ Tellurization of these samples indicates that the conversion to WTe_2_ may be facilitated by the reduction of tungsten bronze.

During the tellurization process, both the WO_3_ nanowires and nanoribbons are converted to the orthorhombic (T_d_) WTe_2_ (lattice parameters: *a* = 6.282 Å, *b* = 3.496 Å, *c* = 14.070 Å), with the conversion evidenced by a peak at 12.5° 2Θ, corresponding to the (002) plane (PDF 01-071-2156 ^28^).

The XRD patterns of WTe_2_ nanoribbons and nanowires indicate that most of the WO_3_ peaks are no longer present, with residual signals at 33.1 and 33.2° 2Θ being of insignificant intensity, demonstrating effective conversion to WTe_2_.

Figure 3a–c summarizes the TEM characterization of the fabricated WTe_2_ nanoribbons. The determined interlayer spacings of ∼0.34 and 0.64 nm confirm the orthorhombic structure, and the nanoribbon growth direction is along the [010] direction (space group *Pnm*2_1_).

**Figure 3:**
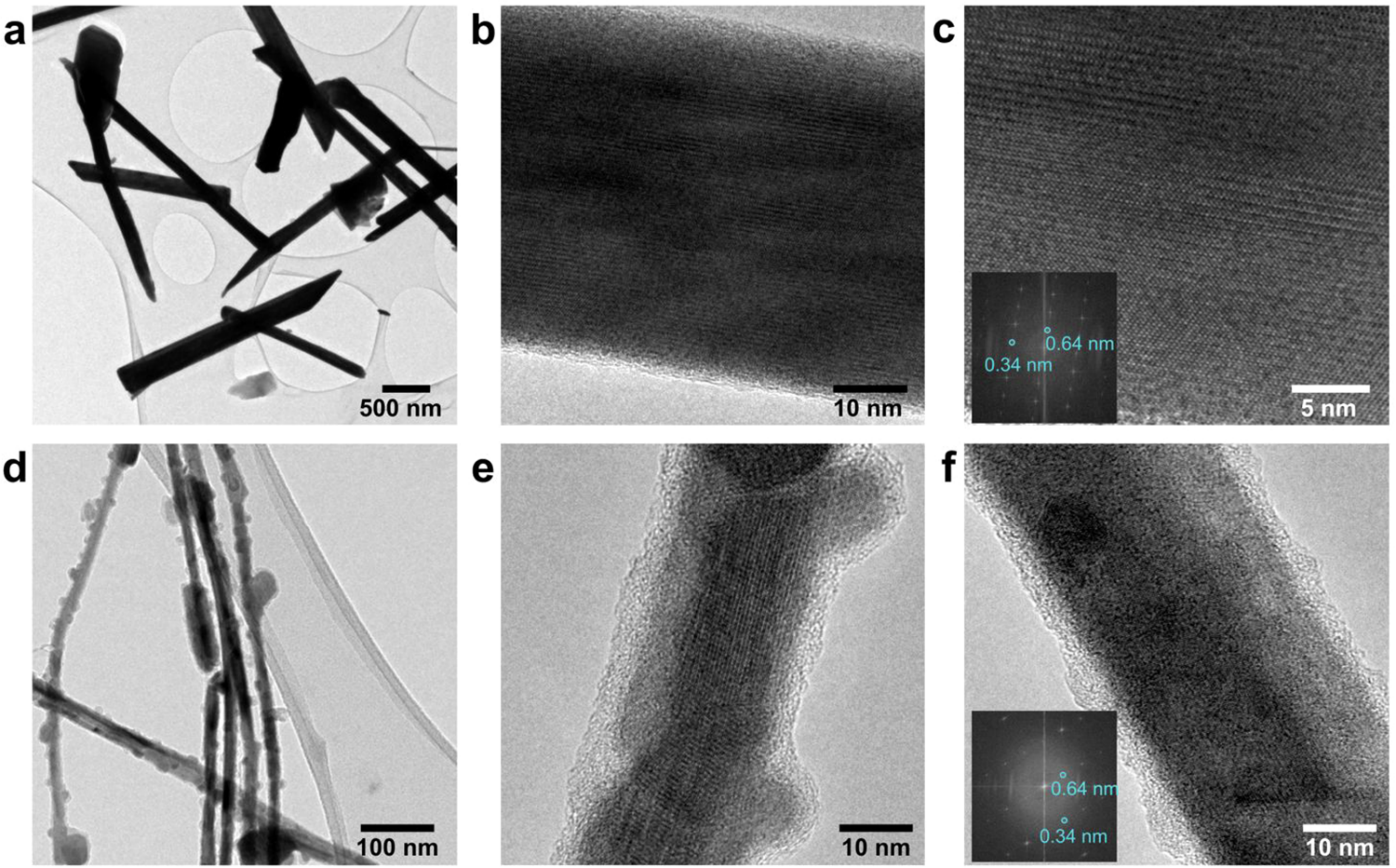
TEM characterization of WTe_2_ nanoribbons and nanowires of the same batch for each growth approach. (a, d) Representative images of WTe_2_ nanoribbons obtained from WO_3_ nanoribbons grown using the WO_3_:NaCl:Te precursor and WTe_2_ nanowires synthesized from WO_3_ nanowires obtained from WO_3_ powder without additives, respectively. (b, c, e, f) TEM images of a WTe_2_ nanoribbon and nanowire, respectively. The insets in (c) and (f) show the fast Fourier transform (FFT) of the corresponding images.

A uniform crystal structure is achieved only for telluride nanoribbons grown from the WO_3_:NaCl:Te precursor (Figure 3c,d, 600 °C, 120 min). The same tellurization configuration (Figure S1c), but with a shorter time of 60 min instead of 120 min for nanowires synthesized from WO_3_, leads to a polycrystalline and disrupted structure, although some homogeneous areas of WTe_2_ can still be found (Figure 3f). The nanowires after the tellurization become decorated with particles, likely of tellurium, with sizes reaching several tens of nanometers. Since the initial WO_3_ nanowires are approximately 100 times narrower than the WO_3_ nanoribbons, perhaps an even shorter tellurization time/lower temperature could be used. However, lower tellurization temperatures (600, 575, 525, 500 °C) and shorter times (40, 20, 15, and 10 min) consistently lead to WTe_2_ nanowires decorated with Te particles (summarized tellurization results are included in the SI, Table S2). At the lowest temperature used (500 °C) and a reaction time of 5 min, no Te particles were observed, but the XRD pattern also showed no WTe_2_ signal (Figure S6).

It is worth noting that the source WO_3_ nanoribbons and nanowires are both highly crystalline, and TEM characterization (SI, Figures S5 and S6) reveals a well-defined tetragonal structure with the growth direction along the *c*-axis, without the presence of any particles. The use of different precursor compositions does not seem to work for obtaining, for example, different phases of WO_3_, as both nanowires and ribbons exhibit the same phase. However, the presence of sodium could alter the tellurization reaction. Sodium is reported to act as a catalyst (forming Na_2_Te), lowering the tellurization temperature to ∼525 °C,^29^ which stabilizes the formation of telluride. Although no extra sodium was added during tellurization, it is present in the WTe_2_ nanoribbon samples fabricated from the WO_3_:NaCl:Te precursor. For the thicker nanoribbons (*t*_NR_ ∼ 200 nm) grown closer to the edge of the Si substrate (SI, Figure S2), EDX spectra show about 0.23 atom % of Na (SI, Figure S3a and Table S2). The presence of sodium and larger nanoribbon dimensions allow the production of good-quality WTe_2_ nanoribbons in the tellurization process. Although the absence of additives in the precursor is expected to lead to WTe_2_ nanostructures of higher quality, our results indicate that it is challenging to produce these nanowires in the tellurization process while maintaining their crystallinity. Reported characteristics of increased electron scattering of WTe_2_ nanowires^12^ could be due to the presence of a polycrystalline structure.

Both WTe_2_ nanowires and nanoribbons were further transferred to SiO_2_ substrates and used to study the charge transport. Figure 4a,b shows optical and SEM images of the fabricated WTe_2_ nanoribbon devices. Two types of devices were measured: as-grown ribbons and those coated with 3 nm of ZnO prior to transfer to SiO_2_ substrates. Longitudinal resistance *R*_*xx*_ measured in a four-electrode configuration for an as-grown and noncapped WTe_2_ nanowire (grown from WO_3_ without additives) is plotted in Figure 4c, device D9_1. The *R*_*xx*_(*T*) shows insulating behavior across the entire temperature range, while the other fabricated nanowire devices in a four-electrode configuration only work at room temperature (see summarized room-temperature resistivity values in Table 1). A similar characteristic is also seen for another device, D5, a ZnO-capped nanoribbon with a much longer distance between the two inner electrodes; thus, the insulator-like *R*(*T*) characteristic is a common feature for both WTe_2_ wires and ribbons. The fact that the ZnO capping layer seemingly fails to prevent rapid oxidation of WTe_2_ for this long ribbon device might suggest an uneven ZnO coverage, not forming a perfect shell. The determined resistivity of the ZnO-capped nanoribbons at a temperature of 2 K as a function of their geometrical parameters does not vary significantly, indicating a similar defect and charge carrier concentration from nanoribbon to nanoribbon. An exception is the widest and longest nanoribbon, D5, which once more points to presumably imperfect coverage of the ZnO capping for this device.

**Figure 4:**
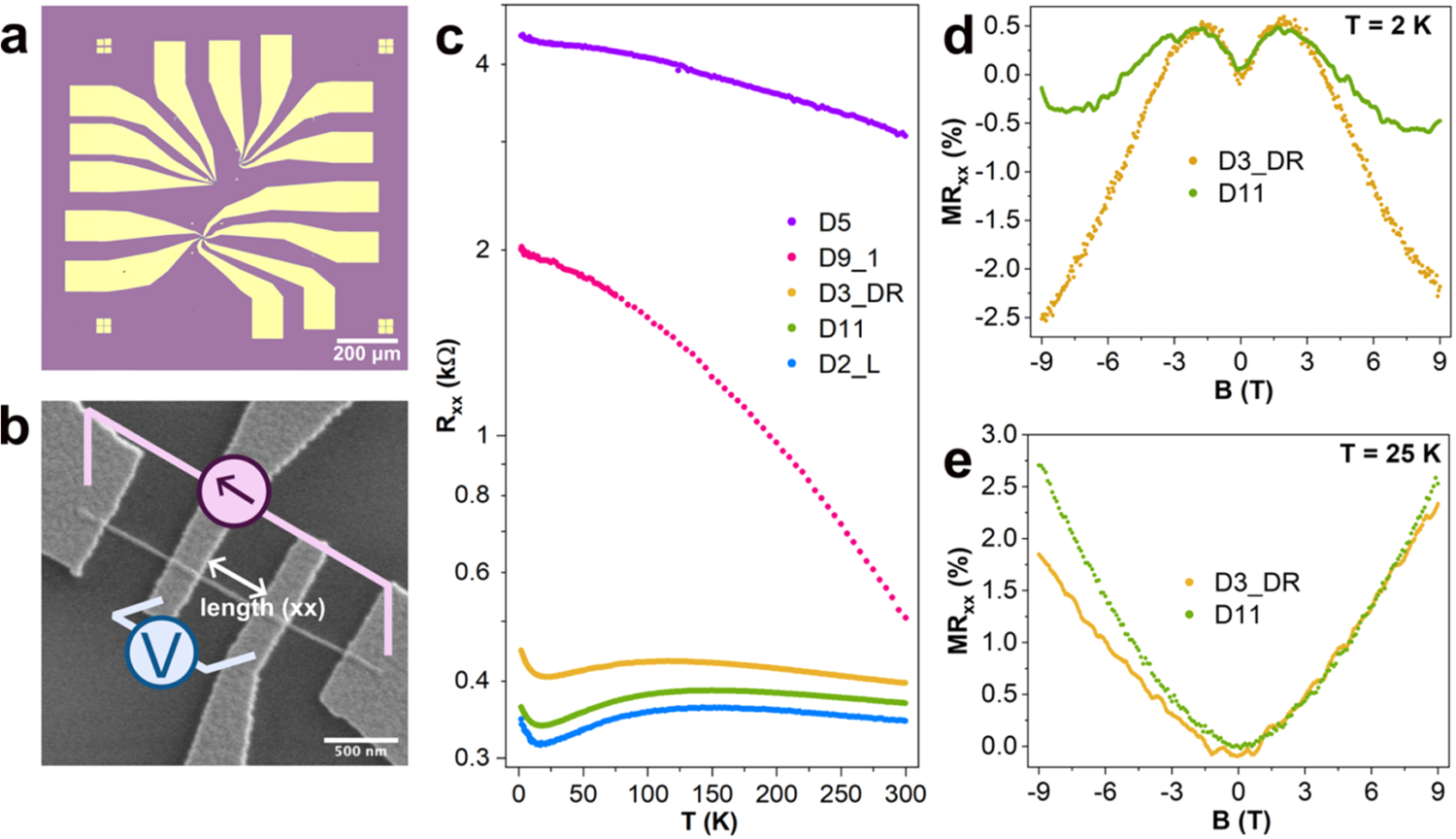
(a) Optical image showing an overview of the nanodevices. (b) SEM image of a WTe_2_ nanowire device for charge transport measurements. (c) Temperature–resistance (*R*_*xx*_) dependence of WTe_2_ nanoribbons and nanowires, measured in a four-point configuration. (d, e) Magnetoresistance MR_*xx*_% = [*R*_*xx*_(*B*) – *R*_*xx*_(0)]/*R*_*xx*_(0) × 100 as a function of magnetic field, measured for two nanoribbons at *T* = 2 and 25 K, respectively. The magnetic field is applied ⊥ to the *a*–*b* plane of the ribbon (∥ to the *c*-axis).

**Table 1:** Determined Resistivity and Dimensions of WTe_2_ Nanowires and Nanoribbons

					ρ, mΩ·cm			
	Device ID	*L_xx_* ^a^, nm	*t*, nm	*w*, nm	2 K	25 K	300 K	capping
WTe_2_ nanoribbons	D6_D	300	130	90	2.5	1.9	1.9	uncapped
	D11	290	50	330	2	1.9	2.1	ZnO
	D2_L	460	112	260	2.2	2.0	2.2	ZnO
	D3_DR	500	150	240	3.3	3.0	2.9	ZnO
	D5	1020	101	410	18	17	12	ZnO
WTe_2_ nanowires	D9_1	350	36	40	0.74	0.70	0.19	uncapped
	D9_2	240	42	60	—	—	2.9	uncapped
	D15_D	180	43	40	—	—	2.8	uncapped
	D13	350	31	40	—	—	2.7	uncapped
	D9_3	260	25	34	—	24	11	ZnO

a Length between longitudinal electrodes (Figure 4b).

Measured *R*_*xx*_(*T*) characteristics of WTe_2_ nanoribbons (grown from WO_3_ with additives, Figure 4c) in the temperature range of ∼100–25 K show metal-like behavior, even for the noncapped nanoribbon D6_D. At temperatures below ∼25 K, an upturn in resistance is observed as the temperature decreases, for both the as-grown (nanoribbon D6_D) and ZnO-capped nanoribbons (devices D11, D2_L, D3_DR). This could be attributed to weak antilocalization,^30^ as the magnetoresistance (MR%) for these nanoribbons at 2 K shows a characteristic WAL feature at low magnetic fields (Figure 4d). The overall shape of the MR­(*B*) curves, however, presents negative magnetoresistance, particularly for the nanoribbon D3_DR at 9 T. This could indicate the chiral anomaly effect, which is predicted for compensated semimetals.^31^ Another plausible and more likely mechanism for the observed negative magnetoresistance is disorder.^32^ At a higher temperature of 25 K, positive magnetoresistance is observed, but at a magnetic field of 9 T, it increases only by ∼2%. Similar suppressed magnetoresistance values for WTe_2_ nanostructures have been reported earlier,^20,33^ due to disorder, presumably induced by the surface oxide, as WTe_2_ is sensitive to air and oxidizes rapidly. In this case, electron–hole compensation in WTe_2_ is preserved, but charge mobilities are greatly reduced.^20,33^ This aspect should be addressed by implementing appropriate nanoribbon encapsulation approaches. Since metal-like *R*_*xx*_(*T*) characteristics were achieved only for the WTe_2_ nanoribbons grown from the WO_3_:NaCl:Te precursor, which most likely present Na impurities, alternative methods for producing WTe_2_ without additives should be explored. However, if one focuses on the tellurization of oxide precursors, it is likely that tellurization catalysts will be required to achieve effective conversion.

## Conclusions

In this work, the synthesis of WO_3_/WTe_2_ nanoribbons was studied using two methods: with and without the NaCl:Te additive. In both approaches, freestanding tetragonal WO_3_ nanoribbons were fabricated, differing only in their geometrical parameters. The subsequent tellurization process for WO_3_ nanoribbons grown with the WO_3_:NaCl:Te precursor yields highly crystalline, metal-like, orthorhombic T_d_ WTe_2_ nanoribbons. In contrast, WO_3_ nanowires grown without additives were challenging to convert to telluride. To address the sensitivity of WTe_2_ to air exposure, ZnO coatings were applied to the nanoribbons, successfully preserving their structure and enabling reliable resistance measurements in a four-electrode configuration.

## Supplementary Material



## Notes

Summary of WTe_2_ nanostructure growth methods (Table S1); schematic representation of nanowire and nanoribbon synthesis setups (Figure S1); characterization of WTe_2_ nanoribbons grown from the NaCl:Te:WO_3_ precursor (SEM, AFM, TEM, EDX, and XRD studies (Figures S2–S5 and Table S2)); characterization of WTe_2_ nanowires grown from WO_3_ powder without additives (SEM, TEM, and XRD studies (Figures S6 and S7 and Table S3)); XPS of WTe_2_ thin-film sample (Figure S8); and SEM images of the fabricated nanoribbon devices (Table S4) (PDF)
