# Supplementary material for: Comparative Studies for the Fabrication of WTe2 Nanoribbons in the Tellurization Process
Source: J Phys Chem C Nanomater Interfaces. 2026 May 11;130(20):7079–85. doi: 10.1021/acs.jpcc.5c08745 (PMC13200692; doi:10.1021/acs.jpcc.5c08745)
Supplement: Supplementary file 1 [file jp5c08745_si_001.pdf]

Supplementary Information: Comparative studies for fabrication of WTe_2 nanoribbons in tellurization process

Jeļena Sušinska[1], Aina Semjonova[2], Reinis Ignatans[3], Gunta Kunakova[1*]

[1]Institute of Chemical Physics, Faculty of Science and Technology, University of Latvia

[2] Department of Chemistry, Faculty of Medicine and Life Sciences, University of Latvia, Riga, Jelgavas St.1, LV-1004, Latvia

[3]Institute of Solid State Physics, University of Latvia, Riga, Ķengaraga St. 8, LV-1063, Latvia

*gunta.kunakova@lu.lv

Table S1. Comparison of WTe_2 nanomaterial (nanobelts, nanowires, nanorods, and nanoribbons) synthesis and structural parameters

	Method		Precursor		Substrate	Tellurization (Growth)			Width, nm	Thickness, nm	Length, µm	Structure	
	WO_x	WTe_2	WO_x	WTe_2		Temperature, °C	Time, min	Carrier gas flow, sccm				WO_x	WTe_2
nanoribbons **(this work)**	2-step CVD		WO_3:NaCl:Te	WO_3 NRs, Te (H_2Te)	Si[100]	600	120	Ar/H_2 80/40	30-2000 (gradient)	30-350 (gradient)	1-50 (gradient)	tetragonal	orthorhombic, T_d
nanowires **(this work)**	2-step CVD		WO_3	WO_3 NRs, Te (H_2Te)	Si[100]	600	60	Ar/H_2 80/40	20-60	20-60	5-10	tetragonal	orthorhombic, T_d
nanowires[1]	2-step CVD		WO_3	WO_3 NWs, Te (H_2Te)	Si[100]	600	40	Ar/H_2 - /100	<50	<50	1-5	tetragonal	monoclinic, 1T'
nanowires[2]	2-step CVD		WO_3	WO_3 NWs, Te (H_2Te)	Carbon cloth	460/550	240	Ar/H_2	<100	~50	>1	monoclinic	orthorhombic, T_d
nanowires[3]	solvo-thermal	CVD	WCl_6, EtOH	$W_{18}O_{49}$ NWs, Te (H_2Te)	deposited on SiO_2/Si for tellurization	600/650	30	Ar/H_2 192/8	15-50	15-50	no info	monoclinic	orthorhombic, T_d
nanorods[4,5]	hydro-thermal	CVD	ammonium metatungstate hydrate, thiourea	WO_3 NRs, Te (H_2Te)	-	650	60	N_2/H_2 15/20	~100	~100	<1	tetragonal	monoclinic, 1T'
nanobelts[6]	-	CVD	-	WCl_6, Te (H_2Te)	SiO_2/Si	700	120	Ar/H_2 200/20	200-500	~100	~10	-	-
nanobelts[7]	-	CVD	-	Cu/W/sub-strate, Te	SiO_2/Si[100] or Al_2O_3[0001]	500	10	-	120-200	~50	1.5-3	-	distorted 1T
nanobelts[8]	-	CVD	-	W/Cu/sub-strate, Te	SiO_2/Si	500	0-120	Ar 500	100-600	5-80	1-400	-	monoclinic, 1T'
nanobelts[9]	-	CVT	-	Te/W/substrate	SiO_2/Si	550	90	inert atmosphere	≤600	≤130	≤10	-	monoclinic, 1T'
nanoribbons[10]	-	CVD	-	Te:W:NaCl, Te (H_2Te)	SiO_2/Si	750	4	Ar/H_2 100/40	~800	~2.5	~50	-	monoclinic, 1T'
atomic wires (WTe)[11]	-	CVD	-	KBr, WO_3, Te	SiO_2/Si or sapphire	635/745	15	N_2/H_2 291/9	no info	no info	~15	-	no info

	Method		Precursor		Substrate	Tellurization (Growth)			Width, nm	Thickness, nm	Length, µm	Structure	
	WO_x	WTe_2	WO_x	WTe_2		Temperature, °C	Time, min	Carrier gas flow, sccm				WO_x	WTe_2
nanoribbons	-	CVD	-	WTe atomic wires, Te		no info	15	N_2/H_2 291/9	<15	~20			
nanoribbons[12]	-	CVD	-	WO_3 thin film	$Si/SiO_2/WO_3$	600	60	Ar/H_2 80/40	100-800	20-60	<10	-	orthorhombic, T_d

1) Growth of WO_3 nanoribbons and conversion to WTe_2

The WO_3 nanoribbons were grown using two approaches – with NaCl : Te additive and without. In the additive method, only one tube furnace's zone was used, where the quartz boat with substrate positioned inclined above the precursor (WO_3:NaCl:Te) was placed (Fig. S1 a). For the non-additive method, the 42 cm long test tube was placed in the furnace so the quartz boat with WO_3 precursor is in the left zone and the Si substrate is downstream in the right zone (Fig. S1 b). The system setup for the tellurization process of both sets of nanoribbons was the same. The substrate with previously obtained WO_3 nanoribbons was placed 5 mm downstream from tellurium powder in the quartz boat, which then was put in the test tube (Fig. S1 c).

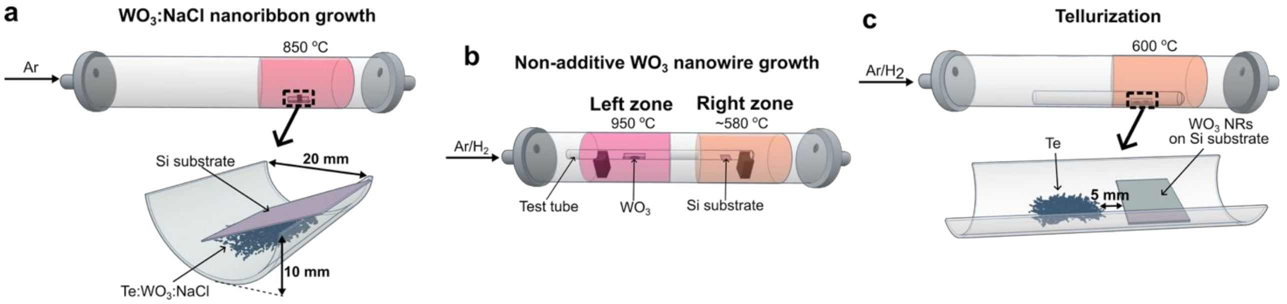

Fig. S1. Schematic representation of two-zone furnace setup for a) growth of WO_3 NRs via Na-additive method; b) growth of WO_3 NRs via non-additive method; c) tellurization process.

<u>2) Characterization of WO_3 / WTe_2 nanoribbons fabricated using NaCl:Te:WO_3 precursor</u>

Due to the Si substrate placement in the WO_3 nanoribbon growth from the Te:WO_3:NaCl source, the dimensions of the nanoribbons vary throughout the sample, forming a distribution gradient with nanoribbons of different thicknesses and lengths. In order to characterize the nanoribbon dimensions, SEM and AFM images were taken at 0.5, 2, 4, ... 16 mm position on the Si substrate. Obtained nanoribbon thickness data are summarized in Fig. S2 (a), and Fig. S2 (b) shows the corresponding SEM images.

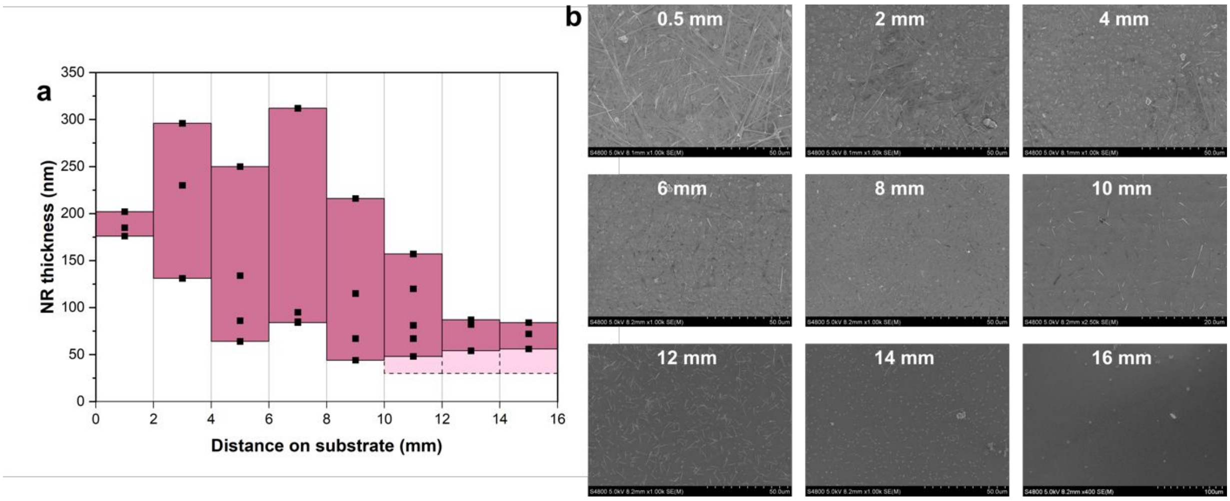

Fig. S2. a) Nanoribbons' thicknesses at different locations on the substrate. Each point refers to a measured nanoribbon; b) SEM images of WTe_2 nanoribbons taken at 0.5 – 16 mm distance on the substrate.

As the WO_3 nanoribbons on the Si substrate are distributed unevenly, this could imply also different distribution of incorporated Na, if any. Therefore element analysis using SEM/EDX of WTe_2 nanoribbons obtained from WO_3 grown using the Te:WO_3:NaCl source were performed, capturing EDX spectra in a point on nanoribbons, that were grown in a certain region (specific distance from the Te:WO_3:NaCl source powder). Obtained atomic ratio at% of detected elements in the EDX spectra are summarized in the Table S1, and suggest that: 1) the quantity of sodium in nanoribbons is not dependent on distance from precursor; 2) the quantity of sodium is very small and in most cases below the detection limit.

Table S2. Atomic ratio (at%) and error (3σ at%) of elements from EDX elemental analysis of WTe_2 nanoribbons obtained via tellurization of Na-additive method WO_3 nanoribbons.

Dist. on substr., mm	W		Te		O		C		Si		Na	
	at%	3σ at%	at%	3σ at%	at%	3σ at%	at%	3σ at%	at%	3σ at%	at%	3σ at%
0.5	4.8	0.6	6.7	0.7	5	3	14	8	69	9	0.23	0.18
2	4.3	0.5	5.8	0.6	5	3	13	7	72	10	0.01	0.08
4	3.0	0.4	4.2	0.4	4	2	12	6	77	10	0	-
6	1.7	0.2	2.0	0.2	4	2	11	6	81	10	0.03	0.08
8	4.4	0.5	6.0	0.6	6	3	10	5	74	10	0	-
10	2.9	0.4	3.9	1.5	6	3	13	9	74	10	0	-
12	1.5	0.2	1.7	0.2	2.8	1.5	13	6	81	8	0.09	0.12
14	2.8	0.4	3.8	0.4	6	3	15	8	72	9	0.01	0.08

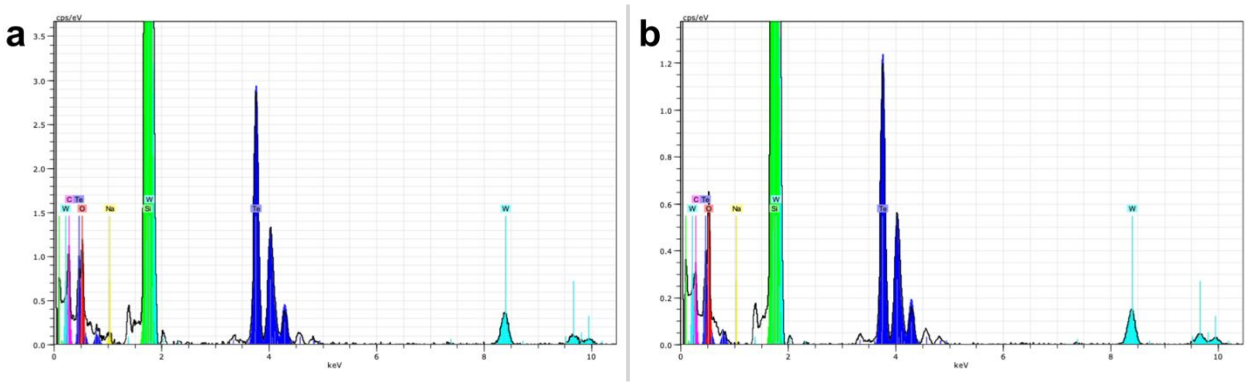

Fig. S3. EDX spectra of WTe_2 nanoribbons (from Na-additive method). Measurement taken in a point of an individual nanoribbon on Si substrate at a) 0.5 mm and b) 8 mm distance on the substrate (with respect from the Te powder), see sample placement configuration in Fig. S1 (a).

Figure S4 (a – c) show TEM images of the WO_3 nanoribbons (grown using the Te:WO_3:NaCl source). The crystallographic structure is tetragonal and determined growth direction is along the c axis.

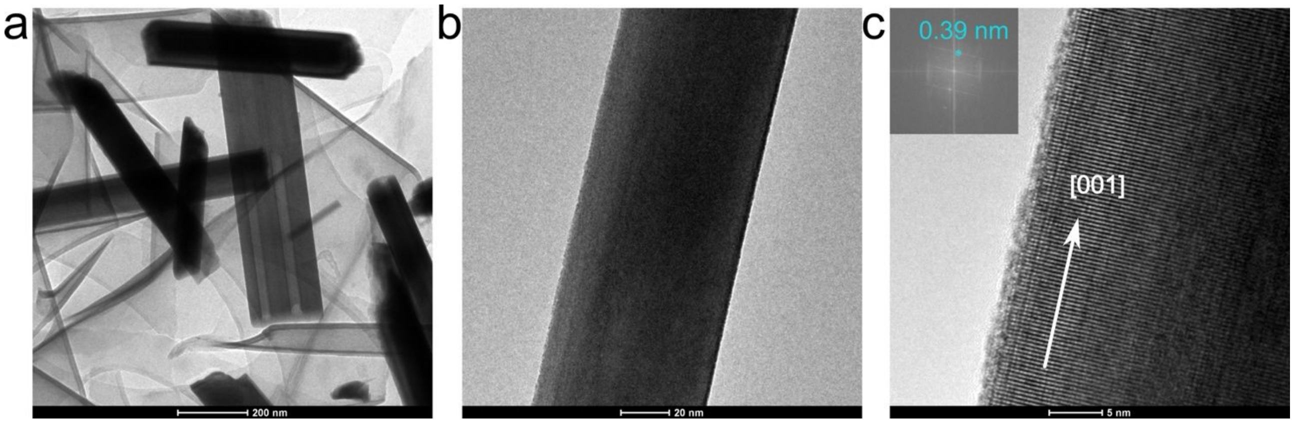

Fig. S4. Structural characterization of WO_3 nanoribbons grown using the Te:WO_3:NaCl source: a) – c) TEM images with a different magnification. In the inset of (c) – fast Fourier transformation (FFT) of the corresponding image.

To understand better the role of incorporated sodium, we conducted additional syntheses using different WO_3:NaCl ratios, and nanoribbon sample with the highest Na content was then used for conversion to WTe_2. The total precursor weight matches that of the standard recipe (equivalent to 70 mg of precursor mixture Te : WO_3 : NaCl = 35:8:1, see methods of the main text), preserving same growth conditions. The sample obtained from WO_3:NaCl 1:1 precursor is then used to evaluate sodium effect on tellurization. X-ray diffraction (XRD) patterns of WO_3 nanoribbons grown with NaCl:WO_3 ratios of 10:1, 8:1, 3:1 and 1:1 are depicted in Fig.S5 All four XRD patterns (Fig.S5 (a)) of WO_3 nanoribbons exhibit the characteristic peaks of sodium tungsten bronze Na_xWO_3 ($x \approx 0.28$ and/or 0.44; PDF 00-048-0222 and 01-075-0240), while stochiometric sodium tungstate Na_2WO_4 likely to be formed as WO_3 reacts with NaCl as $WO_3 + 2\ NaCl \rightarrow Na_2WO_4(l) + WO_2Cl_2\ (g)$ [13] is not detected.

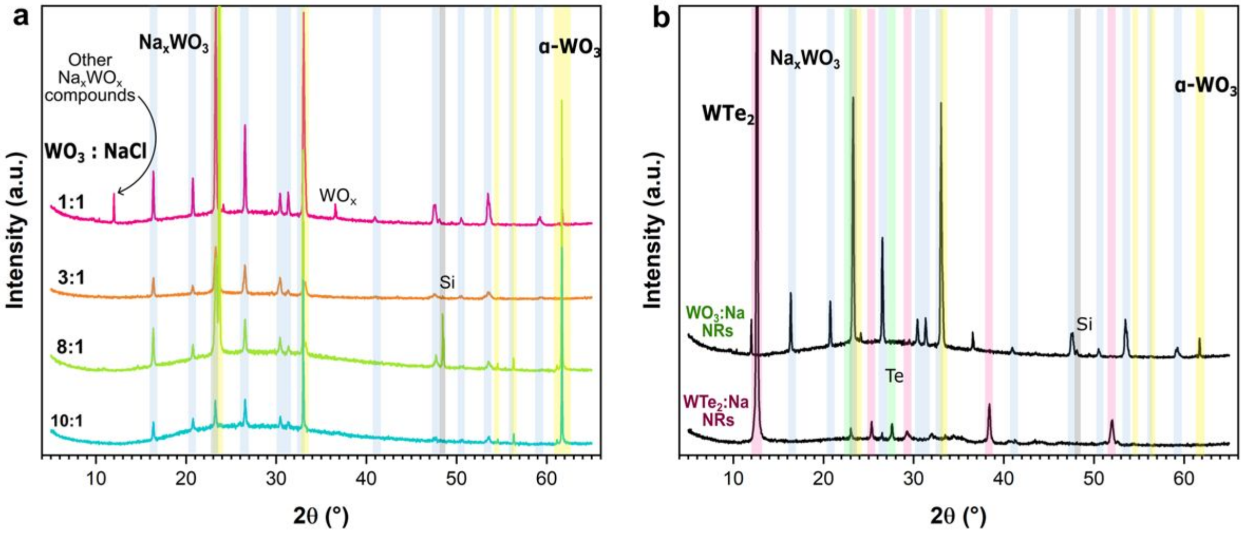

Fig. S5. XRD patterns of (a) WO_3 nanoribbons obtained using different (10:1, 8:1, 3:1 and 1:1) WO_3 : NaCl precursors; (b) WO_3 nanoribbons synthesized using WO_3 : NaCl = 1:1 precursor and corresponding WTe_2 nanoribbons. Blue regions indicate Na_xWO_3 signals, yellow – α-WO_3, pink – WTe_2, green – Te, and grey – Si (substrate).

The relative intensity of the α-WO_3 reflections increase as the NaCl mass fraction in the precursor decreases, indicating that higher NaCl content promotes formation of the sodium – tungsten bronze phase. Absolute peak intensities however cannot be used for quantitative phase analysis as the ribbon areal density on the substrate and local Na incorporation vary across the sample, and random nanowire orientations introduce texture (preferred-orientation) effects that modify reflection intensities. After tellurization of the WO_3:NaCl = 1:1 sample, XRD pattern (Fig. S5 (b)) confirms presence of WTe_2, and almost no Na_xWO_3 signals were observed, indicating that the bronze is conversed (or became XRD-amorphous) during chalcogenization.

Based on the available evidence, one could assume that the reaction between sodium-tungsten bronze (Na_xWO_3) and gaseous H_2Te proceeds through a mechanism closely analogous to that reported for $Na_2WO_4 + H_2Te$ by Rasouli et al. (2019): $Na_2WO_4(l) + 2H_2Te(g) + H_2(g) \rightarrow WTe_2 + 3H_2O(g) + Na_2O$.

3) Characterization of WO_3 / WTe_2 nanowires fabricated using WO_3 powder without additives

Fig. S6 (a – c) summarizes TEM studies of WO_3 nanowires fabricated from WO_3 powder without extra additives. These nanowires possess excellent crystallinity and the same crystallographic structure (and growth direction – along c-axis) as for the WO_3 nanoribbons grown using the Te:WO_3:NaCl.

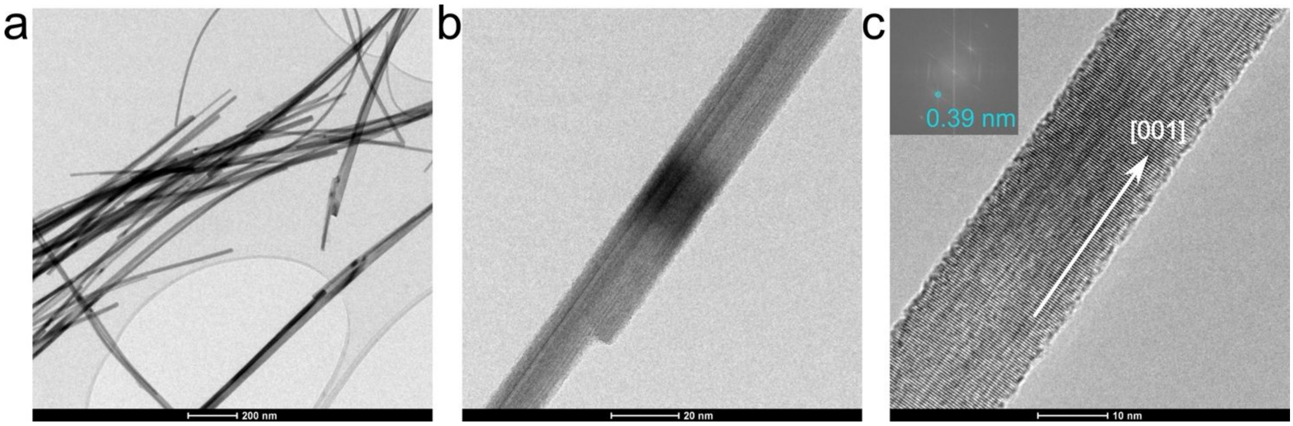

Fig. S6. Structural characterization of WO_3 nanoribbons grown using WO_3 powder without any additives: a) – c) TEM images with a different magnification. In the inset of (c) – fast Fourier transformation (FFT) of the corresponding image.

These nanowires are further used to fabricate WTe_2, in the same tellurization process (see schematics in Fig. S1 c) as the WTe_2 nanoribbons obtained from the WO_3 grown using the Te:WO_3:NaCl source. In this case thin particles likely of metallic tellurium are always present on the nanoribbons. Table S3 summarizes various tellurization parameters and SEM images of the corresponding samples. For all the parameters tested (T=600, 575, 525, 500 ^oC, tellurization time: 120 – 10 min), Te particles with a varying distribution density, is always present on the nanowires. Reducing the tellurization time to 5 min (Table S3, tellurization growth No. 10), the nanowires show no Te particles. However, XRD spectra of this sample (Fig. S7) do not confirm presence of WTe_2.

Table S3. Tellurization parameters of WO_3 nanoribbons obtained via non-additive method, corresponding SEM images.

No.	Temperature, °C	Duration, min	SEM low magnification	SEM high magnification
1	600	60	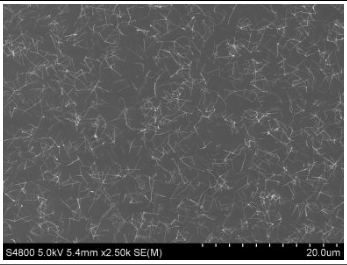	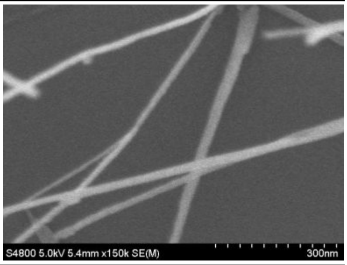
2	575	120	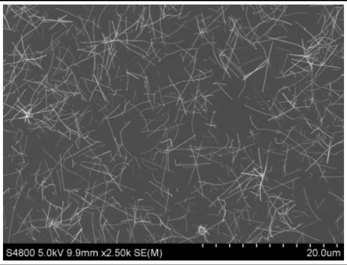	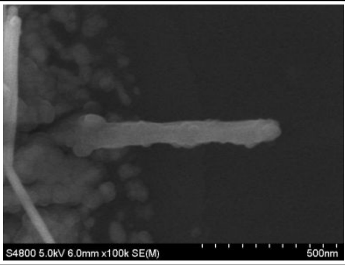
3	575	60	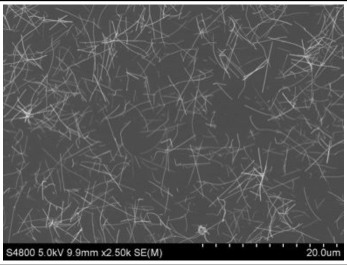	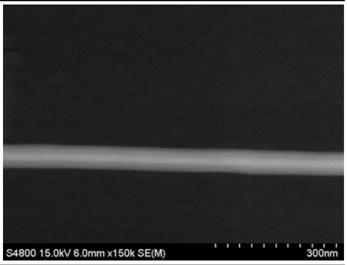
4	575	40	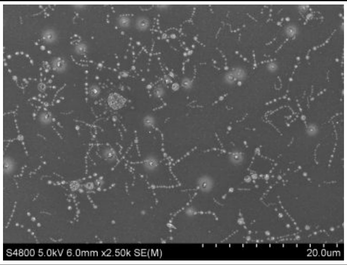	-
5	525	40	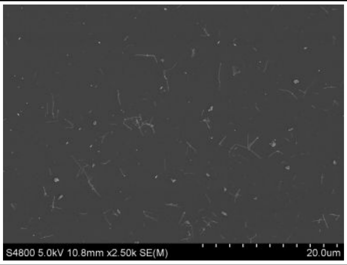	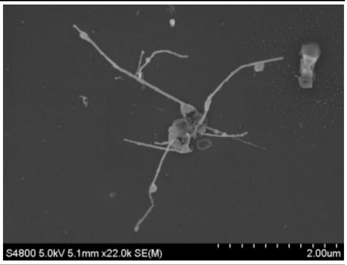
6	525	20	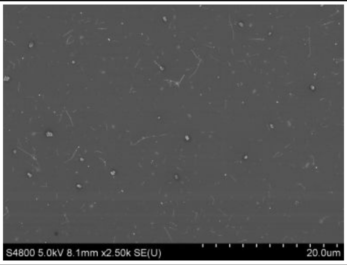	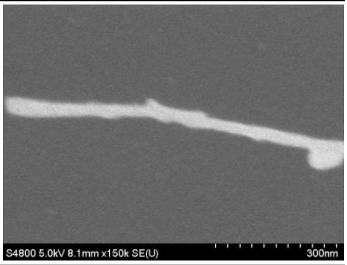

No.	Temperature, °C	Duration, min	SEM low magnification	SEM high magnification
7	525	15	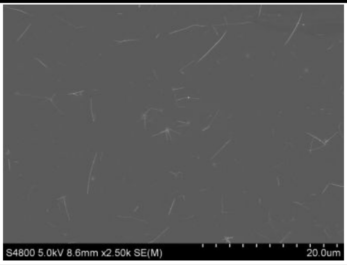	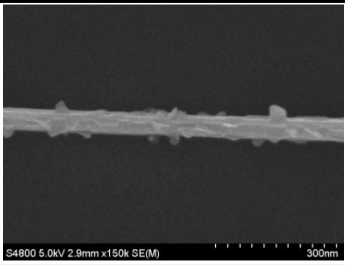
8	525	5	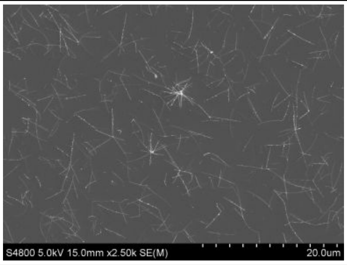	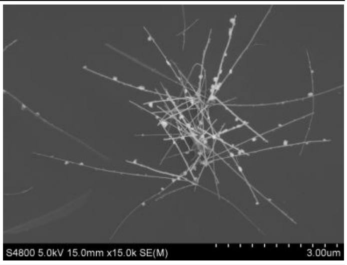
9	500	10	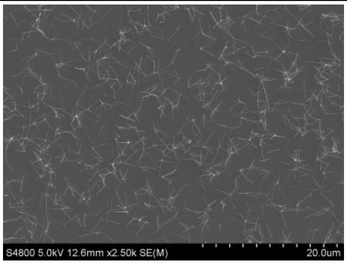	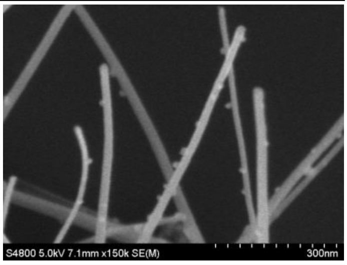
10	500	5	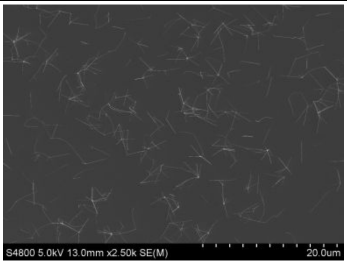	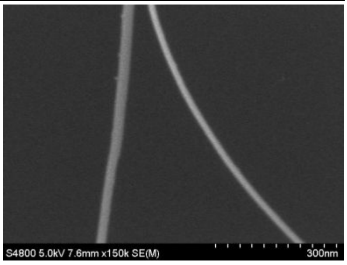

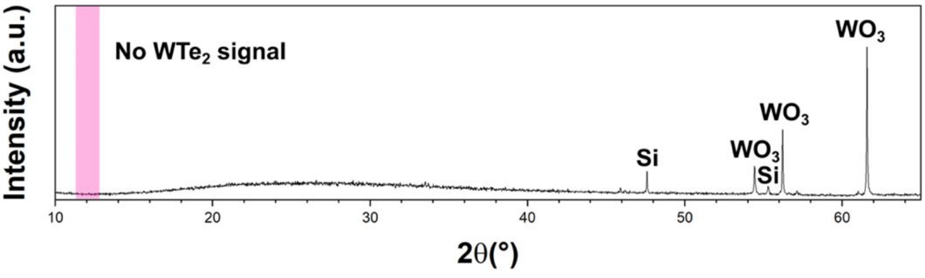

Fig. S7. XRD for WO_3 nanoribbons grown from the WO_3 powder and tellurized at T=500 °C, for 5 min.

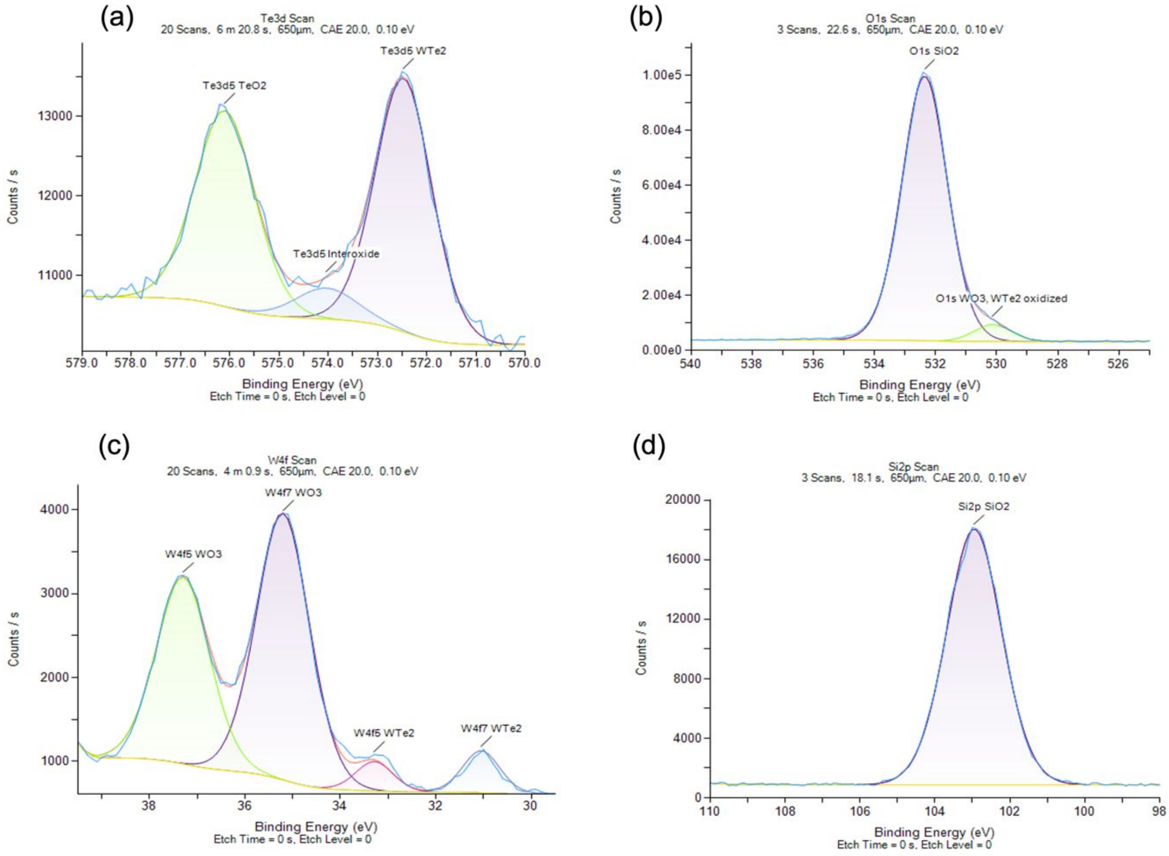

Fig. S8. XPS spectra of WTe_2 thin film synthesized via conversion of WO_3 thin film.

Fig. S8 summarizes XPS studies of the WTe_2 thin film sample, that is prepared via the same tellurization approach as non-additive WTe_2 nanowires. The signals recorded at around 35.0 eV and 37.5 eV represent WO_3 (Fig. S8 (c)). Additional bands at ~31 eV and 33 eV likely represent to WTe_2. The tellurium signals can be seen in Fig. S8 (a), and they indicate on signatures corresponding to TeO_2 (576.0 eV) and also WTe_2 (572.5 eV). These measurements suggest the presence of native surface oxide and correspond with the previous studies on oxidation of WTe_2 surfaces.[14]

4) WTe_2 nanodevices and R(T) characteristics

Table S4. SEM images of the fabricated devices discussed in the main text.

Device ID	SEM image	Device ID	SEM image
D6_D	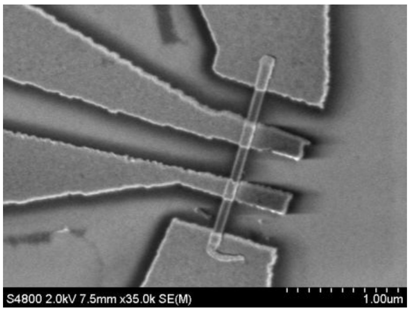	D3_DR	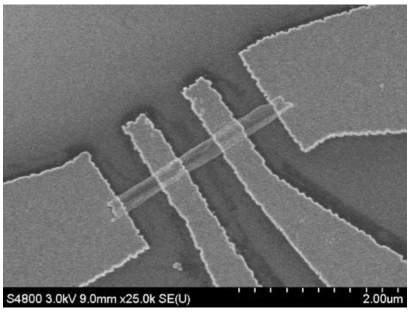
D11	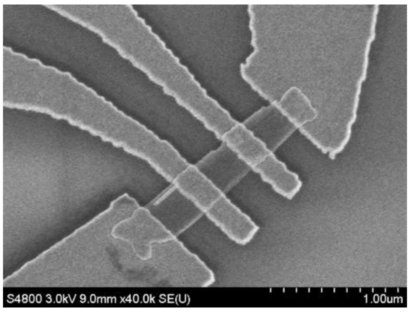	D9_1	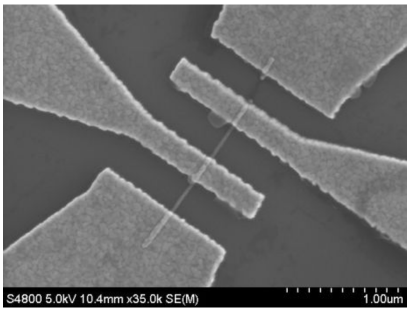
D2_L	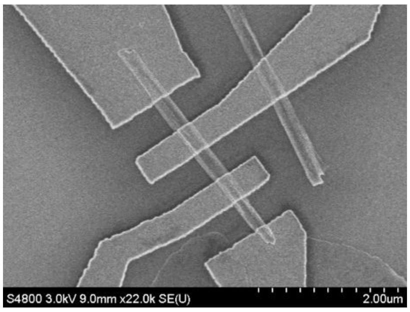		

References

(1) Woods, J. M.; Hynek, D.; Liu, P.; Li, M.; Cha, J. J. Synthesis of WTe2 Nanowires with Increased Electron Scattering. *ACS Nano* **2019**, *13* (6), 6455–6460. https://doi.org/10.1021/acsnano.8b09342.

(2) Kim, H.; Yoo, Y. Active Sites-Enriched Hierarchical Weyl Semimetal WTe_2 Nanowire Arrays for Highly Efficient Hydrogen Evolution Reaction. *Advanced Science* **2025**, *12* (25), 2500516. https://doi.org/10.1002/advs.202500516.

(3) Yomogida, Y.; Nagano, M.; Hamasaki, H.; Hirahara, K.; Miyata, Y.; Yanagi, K. Synthesis of Relatively Small-Diameter Tungsten Ditelluride Nanowires from Solution-Grown Tungsten Oxide Nanowires. *Jpn. J. Appl. Phys.* **2021**, *60* (SC), SCCD02. https://doi.org/10.35848/1347-4065/abe201.

(4) Hong, M.; Li, J.; Zhang, W.; Liu, S.; Chang, H. Semimetallic 1T′ WTe2 Nanorods as Anode Material for the Sodium Ion Battery. *Energy Fuels* **2018**, *32* (5), 6371–6377. https://doi.org/10.1021/acs.energyfuels.8b00454.

(5) Li, J.; Hong, M.; Sun, L.; Zhang, W.; Shu, H.; Chang, H. Enhanced Electrocatalytic Hydrogen Evolution from Large-Scale, Facile-Prepared, Highly Crystalline WTe2 Nanoribbons with Weyl Semimetallic Phase. *ACS Appl. Mater. Interfaces* **2018**, *10* (1), 458–467. https://doi.org/10.1021/acsami.7b13387.

(6) Liu, S.; Qiu, H.; Liu, S.; Zou, J.; Chen, Z. The Study of Atmospheric Pressure CVD Growth Process of MoxW1-xTe2 Nanobelts for Tuneable Chemical Composition. *IOP Conf. Ser.: Mater. Sci. Eng.* **2019**, *678* (1), 012149. https://doi.org/10.1088/1757-899X/678/1/012149.

(7) Kwak, J.; Jo, Y.; Song, S.; Kim, J. H.; Kim, S.-Y.; Lee, J.-U.; Lee, S.; Park, J.; Kim, K.; Lee, G.-D.; Yoo, J.-W.; Kim, S. Y.; Kong, Y.-M.; Lee, G.-H.; Lee, W.-G.; Park, J.; Xu, X.; Cheong, H.; Yoon, E.; Lee, Z.; Kwon,

S.-Y. Single-Crystalline Nanobelts Composed of Transition Metal Ditellurides. *Advanced Materials* **2018**, *30* (30), 1707260. https://doi.org/10.1002/adma.201707260.
(8) Song, S.; Kim, S.-Y.; Kwak, J.; Jo, Y.; Kim, J. H.; Lee, J. H.; Lee, J.-U.; Kim, J. U.; Yun, H. D.; Sim, Y.; Wang, J.; Lee, D. H.; Seok, S.-H.; Kim, T.; Cheong, H.; Lee, Z.; Kwon, S.-Y. Electrically Robust Single-Crystalline WTe2 Nanobelts for Nanoscale Electrical Interconnects. *Advanced Science* **2019**, *6* (3), 1801370. https://doi.org/10.1002/advs.201801370.
(9) Mc Manus, J. B.; Ilhan, C.; Balsamo, B.; Downing, C.; Cullen, C. P.; Stimpel-Lindner, T.; Cunningham, G.; Peters, L.; Jones, L.; Mullarkey, D.; Shvets, I. V.; Duesberg, G. S.; McEvoy, N. Synthesis of Tungsten Ditelluride Thin Films and Highly Crystalline Nanobelts from Pre-Deposited Reactants. *Tungsten* **2020**, *2* (3), 321–334. https://doi.org/10.1007/s42864-020-00056-4.
(10) Xu, M.; Tang, B.; Zhu, C.; Lu, Y.; Zhu, C.; Zheng, L.; Zhang, J.; Han, N.; Guo, Y.; Di, J.; Song, P.; He, Y.; Kang, L.; Zhang, Z.; Zhao, W.; Guan, C.; Wang, X.; Liu, Z. Machine Learning Driven Synthesis of Few-Layered WTe2. arXiv October 10, 2019. https://doi.org/10.48550/arXiv.1910.04603.
(11) Lim, H. E.; Liu, Z.; Kim, J.; Pu, J.; Shimizu, H.; Endo, T.; Nakanishi, Y.; Takenobu, T.; Miyata, Y. Nanowire-to-Nanoribbon Conversion in Transition-Metal Chalcogenides: Implications for One-Dimensional Electronics and Optoelectronics. *ACS Appl. Nano Mater.* **2022**, *5* (2), 1775–1782. https://doi.org/10.1021/acsanm.1c03160.
(12) Amirulloieva, N.; Sušinska, J.; Trimdale-Deksne, A.; Ignatans, R.; Zubkins, M.; Gabrusenoks, J.; Sarakovskis, A.; Kunakova, G. Growth and Magnetotransport Properties of Semi-Metallic WTe2 Nanoribbons. *APL Materials* **2025**, *13* (4), 041128. https://doi.org/10.1063/5.0253094.
(13) Rasouli, H. R.; Mehmood, N.; Çakıroğlu, O.; Kasırga, T. S. Real Time Optical Observation and Control of Atomically Thin Transition Metal Dichalcogenide Synthesis. *Nanoscale* **2019**, *11* (15), 7317–7323. https://doi.org/10.1039/C9NR00614A.
(14) Hou, F.; Zhang, D.; Sharma, P.; Singh, S.; Wu, T.; Seidel, J. Oxidation Kinetics of WTe_2 Surfaces in Different Environments. *ACS Appl. Electron. Mater.* **2020**, *2* (7), 2196–2202. https://doi.org/10.1021/acsaelm.0c00380.
